# Circumferential Pyloric Ulcer Inducing Gastric Outlet Obstruction: A Report of a Rare Case

**DOI:** 10.7759/cureus.71268

**Published:** 2024-10-11

**Authors:** Sourabh Jadhav, Kehkashan Anwar, Ameer Khan, Ilenya Nocivelli, Zuzanna Skolik

**Affiliations:** 1 General Surgery, Tameside General Hospital, Manchester, GBR; 2 Cardiology, Tameside General Hospital, Manchester, GBR; 3 Emergency Medicine, Tameside General Hospital, Manchester, GBR; 4 Obstetrics and Gynaecology, Tameside General Hospital, Manchester, GBR

**Keywords:** abdominal distension, circumferential ulcer, electrolyte imbalance, gastric outlet obstruction, intractable nausea and vomiting, peptic ulcer diseases, upper endoscopy

## Abstract

We present a case of a 46-year-old female who presented to the hospital with significant abdominal distension, intractable vomiting, and pre-renal acute kidney injury (AKI) secondary to hypovolemia. Blood tests revealed global electrolyte derangements and a significant drop in estimated glomerular filtration rate (eGFR) from a baseline of over 90 to 6.7. Diagnostic investigations unveiled a circumferential pyloric ulcer causing gastric outlet obstruction. This case highlights a rare but notable subtype of peptic ulceration leading to this severe clinical presentation, emphasising the importance of early diagnosis and management.

## Introduction

Gastric outlet obstruction (GOO) is a condition whereby there is impaired gastric emptying due to an obstruction in the distal stomach or proximal duodenum. It classically presents with abdominal distension and intractable non-bilious vomiting [1]. These symptoms are often accompanied by epigastric pain, which is exacerbated by eating and often relieved by vomiting. Short-term consequences of GOO include significant electrolyte imbalance and acute kidney injury (AKI). Long-term consequences involve early satiety leading to weight loss and dehydration [2]. If GOO is not managed appropriately, it can become life-threatening for the patient.

The aetiology of GOO can be broadly divided into two categories: malignant and benign. Malignant causes are primarily due to gastric adenocarcinomas, pancreatic cancer, or metastatic disease, which lead to direct luminal obstruction. Benign causes include gastric polyps and peptic ulcer disease (PUD) [1], which was the case in this report. While a common cause of GOO is PUD, circumferential pyloric ulcers are an unusual finding. The exact incidence of circumferential ulcers leading to GOO is not well known due to their rarity [3]. Furthermore, with the advent of effective acid-suppressing therapies, such as proton pump inhibitors (PPIs) and *Helicobacter pylori* eradication therapies, the prevalence of PUD leading to GOO has declined over the years [1]. 

Our case puts a spotlight on this rare subtype of peptic ulcer, leading to a severe manifestation of PUD and GOO.

## Case presentation

A 46-year-old woman was admitted to the hospital with persistent, projectile vomiting, experiencing 20 to 25 episodes per day for two days. She was unable to retain any food, leading to significant weight loss. She did not report any abdominal pain, diarrhoea, fever, or recent travel history; however, her abdomen was markedly distended. Blood tests on admission revealed stage 3 AKI, likely secondary to significant hypovolaemia, with significantly elevated creatinine and urea levels, as well as widespread electrolyte imbalances. Her drug history did not allude to any drugs exacerbating the AKI. Her regular medication included ferrous fumarate, fluoxetine, and levothyroxine. Her past medical history included hypothyroidism and chronic PUD, for which she was also taking omeprazole and famotidine long-term.

The patient was treated in the same-day emergency care (SDEC) centre, where she received IV fluids, antiemetics, and appropriate electrolyte correction. She was discharged with plans to return for repeat urea and electrolyte (U&E) blood tests. Follow-up blood tests continued to show persistent abnormalities, prompting her admission to the hospital under the care of the gastroenterology team for further investigation.

The patient underwent an inpatient oesophagogastroduodenoscopy (OGD) and a computed tomography scan of the thorax, abdomen, and pelvis (CT TAP) to investigate the underlying cause of her symptoms. The OGD revealed esophagitis and gastric ulceration in the pyloric region. However, the scope could not pass beyond the pylorus due to scarring from the ulcer, preventing access to the duodenum. A large volume of dark fluid was found in the stomach, suggesting a gastric bleed, and the patient vomited during the procedure, leading to its abandonment.

**Figure 1:**
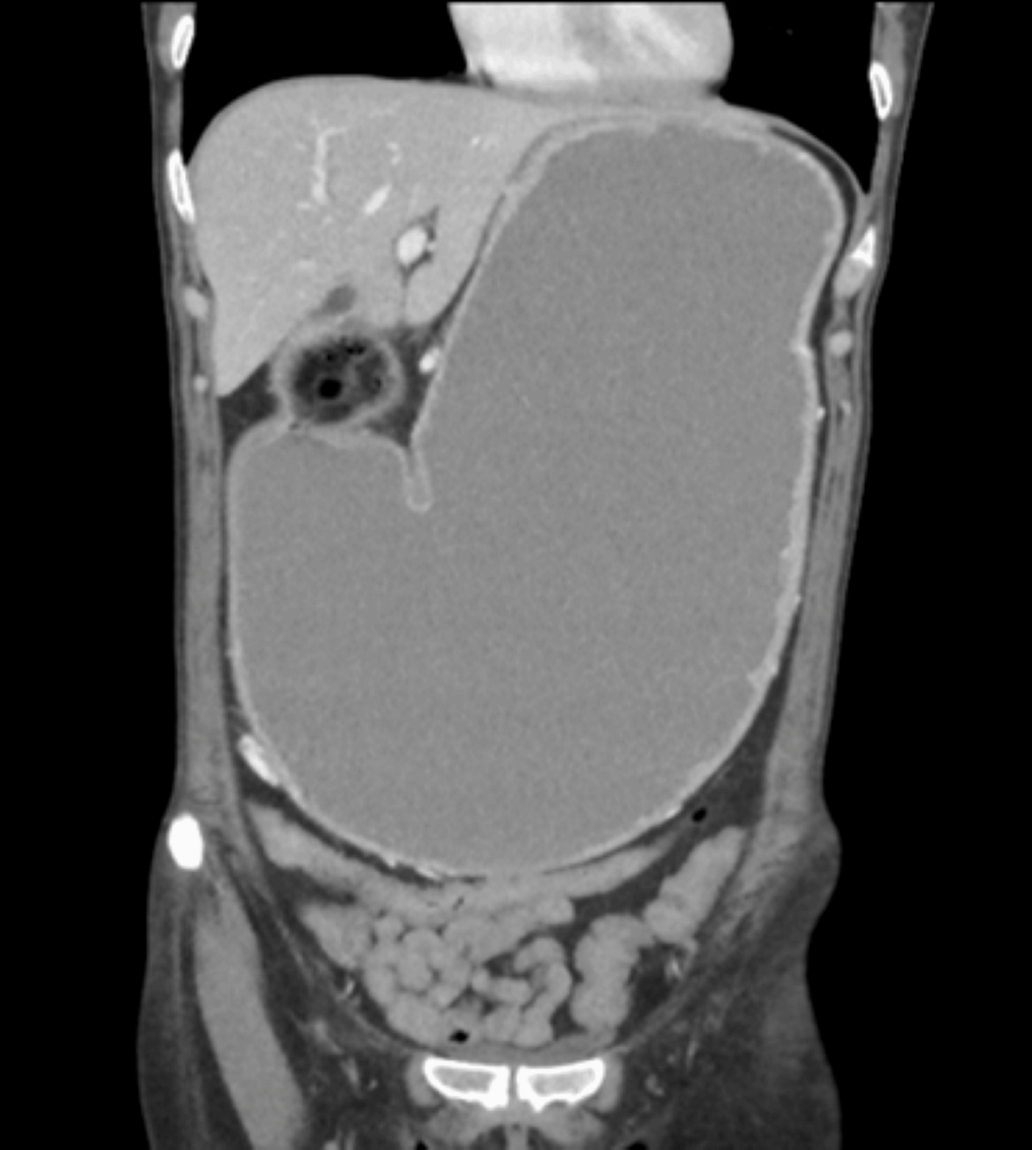
CT scan of the thorax, abdomen, and pelvis in coronal view CT showing marked distension of the stomach, with features highly suggestive of gastric outlet obstruction due to an atypical pyloric circular ulcer with rolled edges. CT: Computed tomography

Following the endoscopy, the patient was started on a high-dose PPI, and a repeat OGD was scheduled for six weeks later. The inpatient CT TAP showed a markedly distended stomach, with the complete collapse of the duodenum, as seen in Figure 1.

The CT report concluded that these features were highly suggestive of GOO, secondary to the narrowing of the pylorus due to an atypical pyloric circumferential ulcer with rolled edges. An urgent discussion with the upper gastrointestinal multidisciplinary team (GI MDT) was advised, and a surgical review was promptly arranged.

**Figure 2:**
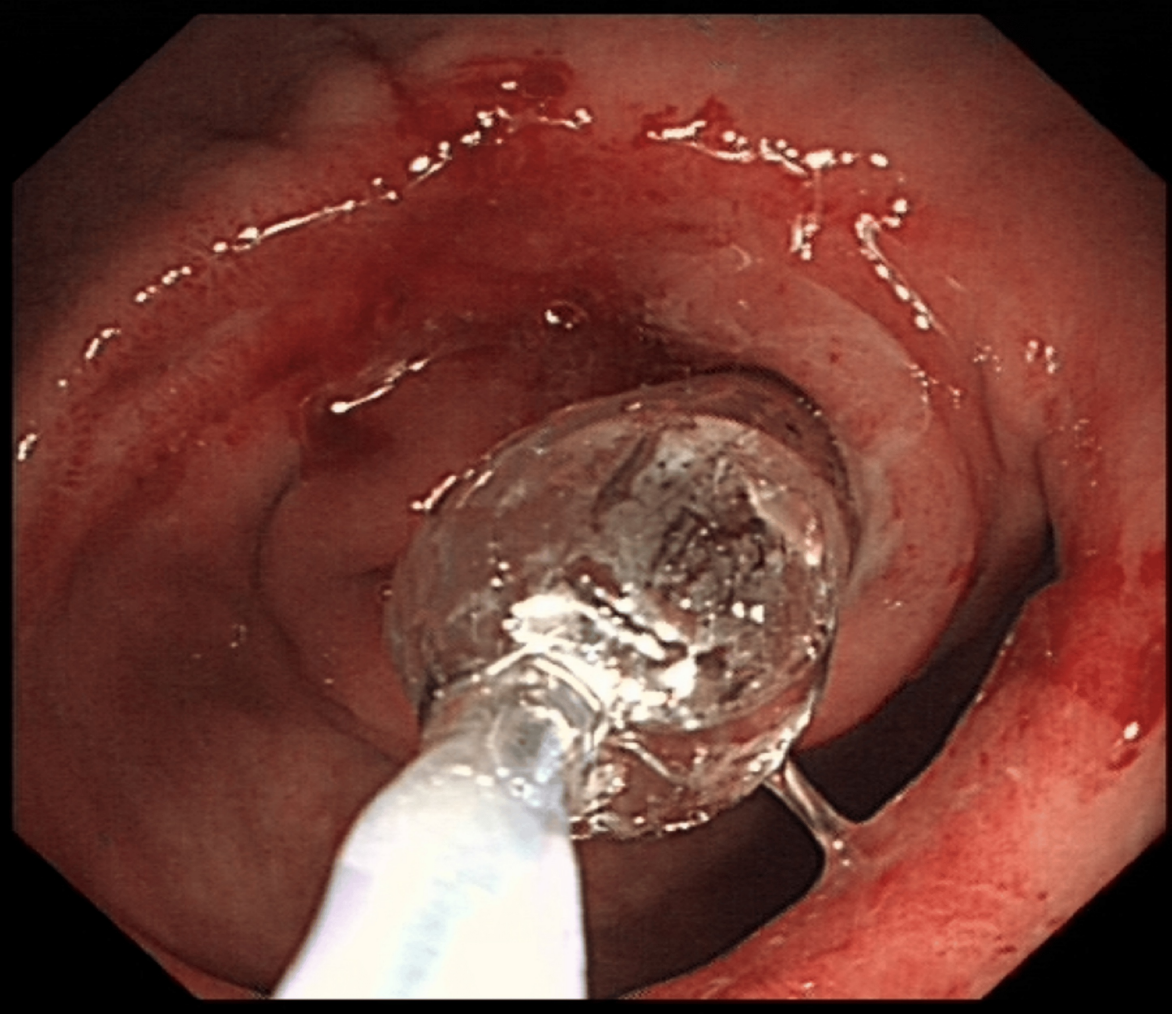
Oesophago-gastro-duodenoscopy (OGD) showing dilation of the pylorus using a balloon dilator

**Figure 3:**
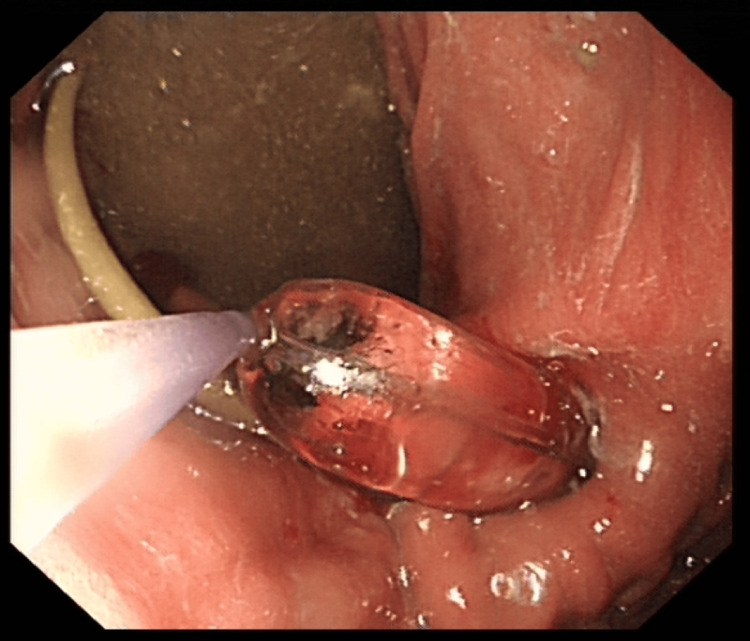
Dilating the pylorus and inserting naso-jejunal (NJ)

The patient's case was reviewed by the upper GI MDT and subsequently by the surgeons. Her care was transferred to the surgical team, and a nasogastric (NG) tube was inserted to relieve the obstruction. She was referred to the dietitians, who recommended naso-jejunal (NJ) tube feeding due to the obstruction at the pylorus. An OGD was scheduled for NJ tube insertion, and total parenteral nutrition (TPN) was started in the meantime. During the procedure, the pylorus was found to be severely narrowed, requiring multiple dilations and multiple attempts at inserting the NJ tube, which can be seen in Figures 2-3, respectively. 

**Figure 4:**
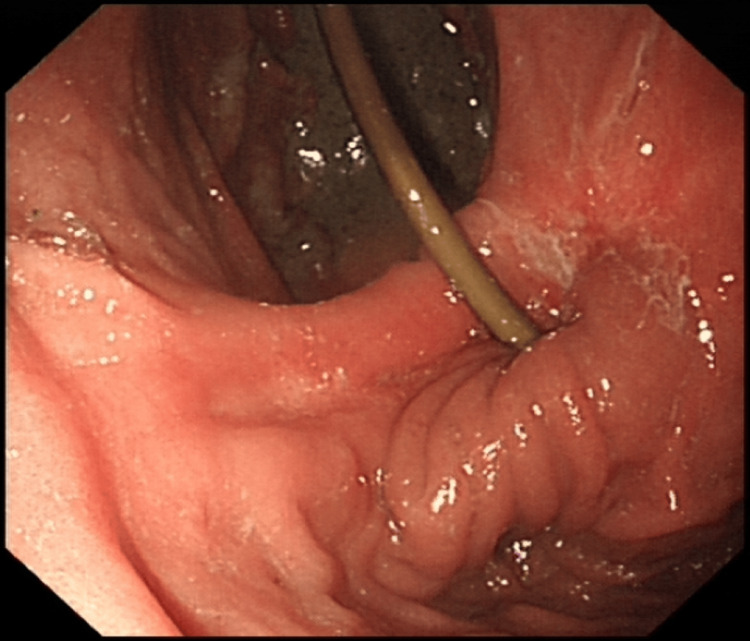
Oesophago-gastro-duodenoscopy (OGD) showing naso-jejunal (NJ) tube successfully inserted into the pylorus

Figure 4 shows the successful insertion of the NJ tube through the stenotic pylorus. After the NJ tube was inserted, the patient began NJ feeding while gradually being weaned off TPN. Her blood tests were monitored regularly, with electrolytes replaced as needed. The patient tolerated NJ feeding well, and her abdominal distension improved while the vomiting subsided. She was educated on proper NJ feeding techniques by the nutrition nurse and was discharged once her electrolyte levels and clinical condition stabilised. The discharge plan included an outpatient abdominal ultrasound to monitor gastric distension, a follow-up OGD with additional pyloric dilation, and a review with the upper GI surgeon two to three weeks post-discharge.

## Discussion

GOO is commonly associated with PUD. Although PUD is a well-known cause of GOO, the development of a circumferential pyloric ulcer leading to obstruction is an uncommon presentation. *H. pylori* is a well-established risk factor for PUD, contributing to chronic inflammation, mucosal damage, and ulcer formation. PUD can lead to gastric GOO through a sequence of pathological events. As PUD progresses, the ulcer leads to chronic inflammation, which extends deeper into the gastric wall. The body responds by forming fibrotic scar tissue around the ulcer, particularly when it occurs in the pyloric region - the narrow passage between the stomach and duodenum. In rare cases, circumferential ulcers can form, causing scar tissue to encircle the entire pyloric canal. As the scar tissue contracts, it narrows the canal, progressively obstructing the gastric outlet [4]. Additionally, ongoing inflammation can cause oedema and further reduce the lumen size, exacerbating the obstruction. This obstruction impairs gastric emptying, leading to symptoms such as persistent vomiting, nausea, abdominal pain, and weight loss. Without intervention, GOO can result in severe complications like dehydration, electrolyte imbalances, and malnutrition [2]. Early diagnosis and intervention are critical to prevent the progression of PUD to GOO.

In cases of GOO caused by benign conditions like PUD, significant pain may not always be present, particularly if there is a chronic history of PUD, as there was in this case. As ulcers heal, scar tissue can develop, leading to a narrowing of the pylorus. This constriction can result in symptoms such as vomiting and bloating [5], without triggering pain, especially if the healing process has already reduced inflammation and irritation in the area. This may explain why, in this particular case, GOO did not present with abdominal pain.

However, the prevalence of GOO caused by PUD has decreased in recent years, largely due to the widespread use of *H. pylori* eradication therapy. The introduction of effective antibiotic therapy targeting *H. pylori*, in conjunction with PPIs, has resulted in a marked decline in the incidence of peptic ulcers and, consequently, complications such as GOO. Studies have shown that the incidence of GOO secondary to PUD has dropped from 5% to 10% of cases in the 1990s to fewer than 2% in populations where *H. pylori* eradication is routine [5].

CT imaging plays a valuable role in the diagnosis and evaluation of GOO, especially when assessing for structural causes such as circumferential pyloric ulcers or malignancies. While endoscopy is typically the primary diagnostic tool for direct visualisation, a contrast-enhanced abdominal CT scan offers a detailed view of the surrounding anatomy and can help assess the severity of the obstruction. It can detect gastric distension, identify the degree of narrowing at the pylorus, and differentiate between benign causes like PUD and malignant obstructions such as gastric cancer [6]. Additionally, CT imaging is useful for identifying complications of GOO, including perforation, abscess formation, and other intra-abdominal pathologies. In emergency settings, CT is often used first to evaluate the extent of obstruction, while endoscopy can confirm the diagnosis via biopsies and provide therapeutic options [7].

Based on the suspected aetiology of GOO, an OGD may be performed following gastric decompression. This procedure can serve as both a diagnostic and potentially therapeutic tool. Endoscopy allows for direct visualisation of the obstruction and facilitates tissue biopsy. Additionally, OGD offers therapeutic potential, with options such as balloon dilation or stent placement to relieve the obstruction [8].

The management of circumferential pyloric ulcer-induced GOO primarily focuses on relieving the obstruction and addressing the underlying cause of the ulcer. Initial treatment involves supportive care, including fluid resuscitation and correction of electrolyte imbalances caused by prolonged vomiting. PPIs are the cornerstone of medical therapy, reducing acid secretion and promoting ulcer healing. In patients with confirmed *H. pylori* infection, eradication therapy with antibiotics is crucial to prevent recurrence [9]. Nutritional support, such as enteral feeding via a NJ tube, may be necessary until gastric emptying improves [10].

When medical therapy alone is insufficient to resolve the obstruction, or when the stricture is severe, more invasive interventions may be required. Endoscopic balloon dilation is one option, allowing for the widening of the narrowed pyloric canal. Success rates vary, with patients often experiencing symptomatic relief; however, repeated dilations may be necessary to maintain long-term patency [11]. When endoscopic interventions fail or the obstruction is too severe, surgical options such as pyloroplasty, gastrojejunostomy, or partial gastrectomy may be considered. Pyloroplasty involves widening the pylorus; gastrojejunostomy bypasses the obstructed region by creating a connection between the stomach and jejunum [12]; and partial gastrectomy involves removing the obstructed portion of the stomach. These procedures are typically reserved for cases where endoscopic management is unsuccessful, or malignancy is suspected [13].

## Conclusions

This case report highlights the rare occurrence of a circumferential pyloric ulcer leading to GOO, a complication that has become less common due to advances in PUD management, particularly with *H. pylori* eradication therapy. The pathophysiological process involving chronic inflammation, fibrosis, and eventual obstruction underscores the importance of early recognition and treatment of PUD to prevent such complications. Prompt diagnosis through endoscopy, followed by appropriate medical or surgical intervention, is essential to improve outcomes in patients presenting with GOO. This case adds to the understanding of rare causes of GOO and reinforces the need for vigilance in atypical presentations.
